# Immune Response Modulation by Vitamin D: Role in Systemic Lupus Erythematosus

**DOI:** 10.3389/fimmu.2015.00513

**Published:** 2015-10-12

**Authors:** Mirentxu Iruretagoyena, Daniela Hirigoyen, Rodrigo Naves, Paula Isabel Burgos

**Affiliations:** ^1^Departamento de Inmunología Clínica y Reumatología, Facultad de Medicina, Pontificia Universidad Católica de Chile, Santiago, Chile; ^2^Programa de Inmunología, Instituto de Ciencias Biomédicas, Facultad de Medicina Universidad de Chile, Santiago, Chile

**Keywords:** vitamin D, systemic lupus erythematosus

## Abstract

Vitamin D plays key roles as a natural immune modulator and has been implicated in the pathophysiology of autoimmune diseases, including systemic lupus erythematosus (SLE). This review presents a summary and analysis of the recent literature regarding immunoregulatory effects of vitamin D as well as its importance in SLE development, clinical severity, and possible effects of supplementation in disease treatment.

## Introduction

1,25-Dihydroxyvitamin D is a steroid hormone, primarily known for its important role in calcium homeostasis (1). The description that several human tissues and cells express the vitamin D receptor (VDR), allows a growing interest in extra-skeletal functions of this vitamin (2). It is now clear that vitamin D plays an essential role in a variety of physiological conditions and that its deficiency is associated with chronic illnesses, including disorders of calcium metabolism, cancers, cardiovascular, and of our special interest, autoimmune diseases: applying to both development and severity of disease (3, 4). In this review, currently available data are summarized to give an overview of the role vitamin D plays on cells of the immune system and the regulation of inflammatory responses, with special emphasis on the role it has in the treatment of systemic lupus erythematosus (SLE).

Vitamin D comes from three potential sources: (i) it can be made in the skin from exposure to sunlight, (ii) nutritional sources, and (iii) supplements (5, 6). In humans, vitamin D is mainly synthesized in the skin after exposure to UVB whereas only a minor part (<10%) is derived from dietary sources (7). There are two major forms of vitamin D: ergocalciferol (vitamin D_2_) that is obtained from UV irradiation and cholecalciferol (vitamin D_3_) that is synthesized in the skin and is present in oil-rich fish (8). Both vitamin D_2_ and vitamin D_3_ are used for food fortification (such as dairy products) and in vitamin D supplements. Vitamin D levels depend on season, reaching their lowest levels after winter and their maximum at the end of summer.

Vitamin D (D represents D_2_, or D_3_, or both) after it is ingested is incorporated into chylomicrons, which are absorbed into the lymphatic system and enter the venous blood. In the skin, cholecalciferol is synthesized from 7-dehydrocholesterol when exposed to UVB. Vitamin D that comes from the skin or diet is biologically inert and requires its first hydroxylation in the liver by the vitamin D-25-hydroxylase (25-OHase) to 25(OH)D, which represents the main circulating vitamin D metabolite and is the most reliable parameter to define human vitamin D status (9). However, 25(OH)D requires a further hydroxylation in the kidneys by the 25(OH)D-1-OHase (CYP27B1) to form the biologically active form of vitamin D 1,25(OH)2D (9). This process is under strict control of parathyroid hormone and the phosphaturic hormone fibroblast growth factor 23 (FGF-23). High levels of vitamin D inhibit CYP27B1 and stimulate CYP24A1, an enzyme that metabolizes vitamin D into the inactive, water-soluble form, calcitroic acid, which is then excreted into the bile. Circulating levels of 1,25(OH)2D are determined by renal CYP27B1 activity. Interestingly, other cell types, including immune cells, also express CYP27B1, and these cells are able to convert the inactive hormone into the active form, in an autocrine or paracrine manner. This process lacks feedback mechanisms (as the ones described for kidney cells), and allows the production of high local concentrations of vitamin D.

1,25(OH)2D interacts with VDR, which is present in several human tissues and cells (1, 9). VDR is a member of transcription factor family, characterized by a highly conserved DNA-binding domain and a structurally conserved ligand-binding domain, and it acts as a modulator of gene transcription (1). 1,25(OH)2D may be responsible for regulating up to 200 genes that may facilitate many of the pleiotropic health benefits that have been reported for vitamin D (1, 5, 6). Ligand binding initiates a conformational change that increases the receptor’s affinity to the retinoid X receptor (RXR), then VDR-vitamin D complex forms heterodimers with RXR and the complex binds to vitamin D response elements on DNA and recruits a number of nuclear co-activator and co-repressor proteins (10). The gene encoding VDR is located on chromosome 12q13.11, contains 9 exons and 8 introns and several single nucleotide polymorphisms (SNPs) have been described. Mainly four, including *Bsm*I and ApaI (both in intron 8), Fok1 and *Taq*I (located in the start codon), have been intensively studied (11).

Serum 25(OH)D is considered as the most accurate marker for vitamin D. Vitamin D deficiency has been recently recommended by the Institute of Medicine (IOM) as a vitamin D of <20 ng/mL, whereas vitamin D insufficiency has been defined as levels between 21 and 29 ng/mL (12, 13). This classification is based on vitamin D effects on bone and mineral homeostasis. The serum concentration of the active 1,25(OH)2D is approximately 1000-fold lower and far below the effective concentration described in *in vitro* studies. Most *in vitro* studies use more that 100-fold higher concentrations of 1,25(OH)2D than found in serum, to obtain an effect. It has been suggested that the level of circulating 1,25(OH)2D is too low to affect immune responses *in vivo*, and that sufficient levels are obtained by local conversion of 25(OH)D3 to 1,25(OH)2D. Other important players influencing the bioavailable levels of vitamin D are the vitamin D-binding protein (DBP) and albumin. 25(OH)D3 and 1,25(OH)2D circulate bound to DBP (85–90%) and albumin (10–15%) with <1% in their free form (14). Studies in mice lacking DBP have shown that DBP acts as a vitamin D reservoir by protecting 25(OH)D3 and 1,25(OH)2D from degradation and renal secretion (15).

The major cause of vitamin D deficiency is inadequate exposure to sunlight (6, 16). There is an inverse association of serum vitamin D and body mass index (BMI), and thus, obesity is also associated with vitamin D deficiency (17). Patients with fat malabsorption syndromes and bariatric patients are often unable to absorb the fat-soluble vitamin D, and patients with nephritic syndrome lose 25(OH)D bound to vitamin DBP in the urine (18). Patients on a wide variety of medications, chronic granuloma-forming disorders, some lymphomas, and primary hyperparathyroidism have a high risk for vitamin D deficiency (19).

Supplementation with vitamin D in the general population has shown fracture prevention, suggested benefit in cardiovascular health, colorectal cancer prevention and reduction of proteinuria in patients with chronic kidney disease (20, 21). However, a possible harm of vitamin D supplementation has been documented in some studies: a meta-analysis showed that supplementation with calcium and vitamin D could be associated with the modest increase in the risk of cardiovascular events, especially myocardial infarction (22). Currently, no international consensus is available on the optimal vitamin D supplementation level; recommendations differ in many countries and medical societies. The Endocrine Society considers a supplementation of 10,000 IU daily to be safe, the IOM considers 4000 IU/day and the European Food and Safety Authority recommends staying below 4000 IU/day. The most common forms of vitamin D for supplementation are cholecalciferol (vitamin D_3_) and ergocalciferol (vitamin D_2_), although administration of calcitriol is limited because of potential side effects. A recent work by Souberbielle et al. (23) has shown that a target level of at least 30–40 ng/mL of vitamin D serum level was recommended in adult patients with risk of fractures, falls, cancers, and autoimmune and cardiovascular disease. Serum levels higher than 150 ng/mL may cause acute vitamin D intoxication with hypercalcemia, hypercalciuria, and calcifications in different organs.

## Vitamin D and the Immune Response

Several studies suggest that calcitriol can enhance the innate immune response, whereas it can inhibit the adaptive immune response (24). Early evidence suggesting that vitamin D could act as stimulant for innate immunity comes from reports about tuberculosis treatment with cod liver oil (25). It enhances chemotaxis and phagocytic capabilities of innate immune cells and activates the transcription of antimicrobial peptides, such as defensin B and cathelicidin (26). Low 25(OH)D concentrations have been linked to increased mortality caused by severe infections in end-stage renal disease patients, and have been associated with upper respiratory tract infections and allergic asthma.

In particular, dendritic cells (DCs) are important targets for the immunomodulatory effects of vitamin D. DCs are professional antigen presenting cells (APCs) that play an important role in maintaining peripheral tolerance by preventing self-reactive T cells from causing autoimmune damage. Through their unique ability to efficiently capture antigens and trigger the adaptive immune response, DCs are critical for the defense against infectious agents and tumors (27). In addition to activating immune responses, DCs also play a central role in peripheral T cell tolerance, by inducing T cell anergy or unresponsiveness to self-antigens (28). Calcitriol and its analogs are able to suppress DC differentiation (29) and maturation *in vitro*. Likewise, vitamin D, by inhibiting the maturation of DCs, can make them tolerogenic (30–32). It has been shown that DC can produce 1,25(OH)2D from 25(OH)D *in vitro*, and respond to this through the VDR in an autocrine fashion. Since DCs are central to the maintenance of self-tolerance, it is possible that a deficiency in vitamin D could have consequences on their maturation and function and consequently on the risk of developing autoimmune diseases as well as disease severity. In addition, vitamin D exerts effects that oppose the effect of IL-4 on MHC class II antigen expression in human monocytes and specifically modulates human monocyte phenotype and function by altering HLA-DR expression and antigen presentation, leaving lytic function intact (33).

**Figure 1:**
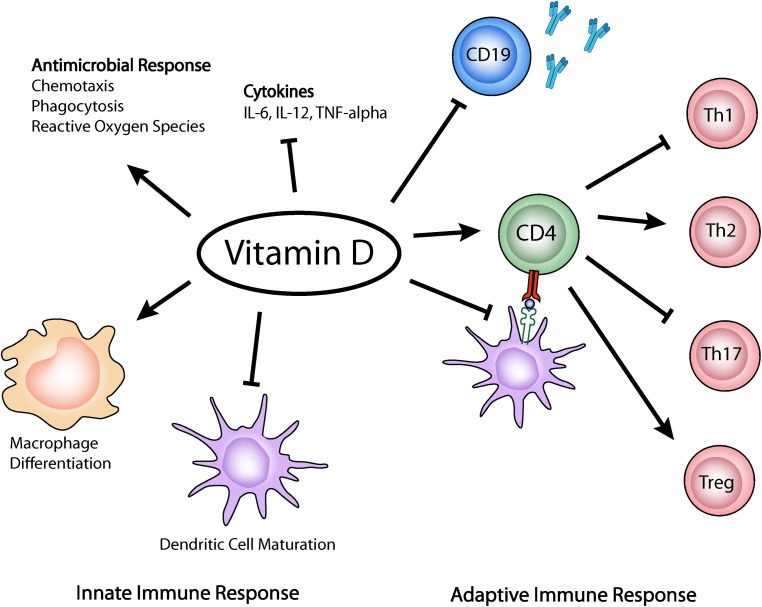
**Vitamin D effects on the innate and adaptive immune response**. Vitamin D has been shown to enhance chemotaxis, antimicrobial peptides, and macrophage differentiation. It can also inhibit DCs maturation, Th1 and Th17 differentiation, and promotes immunoregulatory functions of Treg cells.

T and B cells express VDR and are important target cells of calcitriol immune regulation. Vitamin D can suppress cellular and humoral immunity in several animal models as it plays an important role in regulating proliferation, differentiation of activated B cell, and immunoglobulin production (34, 35). *In vitro* studies have shown that vitamin D inhibits IL-17 synthesis, inhibiting Th17 differentiation and increases the quantity of CD4^+^ CD25^+^ T regulatory cells, which produce IL-10 and amplifies a Th1–Tr1 switch (36) (Figure 1).

## Vitamin D and Autoimmune Diseases

Several studies have now reported vitamin D insufficiencies in various autoimmune disorders. In addition to observational studies, numerous randomized trials have addressed the question whether vitamin D levels are associated with the risk of developing autoimmunity and whether development and disease progression can be influenced by vitamin D supplementation. In the following section, we have summarized the latest results associating vitamin D insufficiency and vitamin D supplementation in SLE. If causal, these associations might be of great importance for public health.

### Vitamin D and systemic lupus erythematosus

Systemic lupus erythematosus is a chronic multisystem autoimmune disease that can manifest with a diverse array of clinical symptoms and which is characterized by the production of autoantibodies directed against nuclear antigens (37). Systemic injury may arise as a consequence of inflammation caused by direct autoantibody-mediated tissue injury and the deposition of complement-fixing immune complexes (ICs) (38). IC-mediated inflammation has been shown to damage multiple organs, such as skin, joints, kidneys, brain, and blood vessels. Cellular and molecular mechanisms underlying this autoimmune disease are not completely understood. Currently, there is no cure for SLE, and treatments, such as long-term corticosteroids, may contribute to further health risks.

In SLE animal models, it has been shown a relation between vitamin D and disease manifestations. Lemire et al. (39) showed that in the MRL/1 SLE mouse model, supplementation with 25(OH)D for 18 weeks reduced dermatologic lesions, proteinuria, and anti-DNA antibodies. Instead, Vaisberg et al. (40) described opposing results in the NZB/W mice that were injected with different concentrations of vitamin D. Treatment with cholecalciferol led to a worsening of the histopathological findings in the kidneys of female F1 NZB/W mice. *In vitro* studies of SLE-derived PBMCs have shown that when these cells are incubated with calcitriol, reduced cellular proliferation and anti-DNA antibodies are observed (41). Also, *in vitro* vitamin D reduced the expression of CD40, MHC class II, CD86, and inhibited the activation of APCs derived from SLE patients (42). Ben-Zvi et al. (43) showed that vitamin D treatment reduced the expression of IFNα-regulated genes in healthy and SLE patients-derived DCs in response to factors in activating SLE plasma.

Levels of micro RNA (miRNA)-146a in PBMCs correlate significantly with disease activity in SLE patients and urinary expression correlates with estimated glomerular filtration rate (44). Recent studies have shown that after treatment with vitamin D, miRNA-146a levels of SLE patients tend to decrease (44).

Association studies with VDR polymorphism and SLE susceptibility have been performed in different populations with controversial results (45–47). A meta-analysis study, including a total of 11 case-control studies (8 from Asian, 2 European, 1 Latino population), of 1683 patients and 1883 healthy individuals revealed associations between the VDR polymorphisms and SLE (48). These findings show that the *Bsm*I and *Fok*I polymorphisms are associated with increased risk of SLE, especially in the Asian population. Limitations to the study are the low representation of European and Latino populations.

Hypovitaminosis D is highly prevalent in SLE as a result of avoidance of sunshine, renal insufficiency, and the use of medications, such as glucocorticoids, anticonvulsants, and antimalarials, which alter the metabolism of vitamin D, vitamin D-binding protein levels or downregulate the functions of the VDR (49, 50). Several studies have reported suboptimal vitamin D levels in patients with SLE, whose prevalence described varies between 36.8% and 75% in the different populations studied (49, 51–54). Low levels of vitamin D have been correlated with disease activity, and are associated with osteoporosis, fatigue, and certain cardiovascular risk factors in SLE patients (49, 55). The wide variation reported can be related to age, ethnicity, geographic location, and season at the time of the study.

### Vitamin D deficiency and disease activity

There are several cross-sectional studies examining the relationship between low vitamin D levels and SLE activity. It has been shown that vitamin D deficiency has an association with disease activity and some clinical manifestations, but there are discrepancies between the different populations studied. No associations between adolescent dietary vitamin D intake and adult SLE risk were observed in a prospective cohort of women (56, 57). Mok et al. reported in a cross-sectional study with 290 Chinese patients with SLE, that vitamin D deficiency was inversely correlated with disease activity, measured by SLEDAI scores (58), and in another study, they reported associations with anti-dsDNA levels (59). In addition, Lertratanakul et al. showed that lower baseline vitamin D levels are associated with higher cardiovascular risk factors and more active SLE (60). Besides, SLE patients with higher vitamin D levels were less likely to have hypertension and hyperlipidemia. A recent study that evaluated 129 Indian patients with SLE found a negative correlation of vitamin D levels with disease activity, anti-dsDNA, plasma IFN-α, and IFN-α gene expression (61). Baseline vitamin D levels were not associated with relapse-free survival rate (62). In Australian patients, Yap et al. showed that low vitamin D is associated with higher disease activity, and an increase in vitamin D was associated with reduced disease activity over time (50). Other studies have shown that vitamin D deficiency is associated with a higher B cell activation, more frequent leukopenia or renal involvement with proteinuria, and higher titers of anti-DNA (51, 63–66). By contrast, others studies have reported no association between vitamin D and fatigue, SLEDAI score or cytokine profile (67–69).

Overall, both *in vitro* and *in vivo* studies of vitamin D effects in SLE provide immunological basis for potential beneficial effects of vitamin D in this disease. Despite all this overwhelming evidence favoring the use of vitamin D in SLE, to date, vitamin D supplementation is not the standard of care for patients with SLE. Interventional studies have been reported (see Table 1), with the purpose of changing clinical outcomes; however, results are still not conclusive.

**Table 1:** **Clinical studies of the effects of vitamin D supplementation in patients with systemic lupus erythematosus**.

Author, year, country	Sample size	Subjects	Intervention (type, dose, duration)	Study results
Petri, 2013, USA (64)	1006	SLE	Oral cholecalciferol 50,000 IU weekly + 200 U calcium/vitamin D twice daily	A 20-U increase in the 25(OH)D level was associated with decrease of 0.22 of SELENA-SLEDAI and 2% decrease in urine protein-to-creatinine ratio
Abou-Raya, 2013, Egypt (71)	267	SLE	Oral cholecalciferol 2000 IU/day or placebo for 12 months	69% suboptimal VitD
				39% deficient VitD
				Lower VitD correlates with higher disease activity
				Change in 25(OH)D after 12 months is associated with improvement in inflammatory-hemostatic markers
Ruiz-Irastorza, 2010, Spain (70)	80	SLE	Oral cholecalciferol 600–800 IU day for 24 months	Beneficial effect on fatigue, no significant correlations were seen in SLEDAI or SDI values
Lima, 2015, Brazil	60	juvSLE	Oral cholecalciferol 50,000 IU/week or placebo for 24 weeks	Beneficial effect on fatigue and decrease disease activity
Aranow, 2015, USA (73)	57	SLE	Oral cholecalciferol 2000 or 4000 IU for 12 weeks	No changes in IFN signature in vitamin D deficient SLE patients
Andreoli, 2015, Italy (72)	34	SLE	Oral cholecalciferol	Intensive regimen significantly raise vitamin D serum levels
			Intensive R: 300,000 IU initial bolus followed by 50,000 IU monthly (850,000 annually)	No significant differences in disease activity, or SLE serology were found
			Standard R: 25,000 IU monthly (300,000 annually) for 12 months and then switched in the second year	
Piantoni, 2015, Italy (74)	34	SLE	Oral cholecalciferol	Vitamin D treatment promotes regulatory T cells proliferation and production of Th2 cytokines
			Intensive R: 300,000 IU initial bolus followed by 50,000 IU monthly (850,000 annually)	
			Standard R: 25,000 IU monthly (300,000 annually) for 12 months and then switched in the second year	
Terrier, 2012, France (75)	20	SLE	Oral cholecalciferol	Increase of naive CD4 T cells
			100,000 IU/week during 4 weeks	Increase in regulatory T cells
			100,000 IU/month for 6 months	Decrease Th1 and Th17 cells
				Decrease memory B cells
				Decrease anti-DNA antibodies

Ruiz-Irastorza et al. showed no significant correlations with SLE clinical activity, evaluating SLEDAI or SDI values, and suggested that increasing vitamin D levels may have a beneficial effect on fatigue (70). Petri et al. studied a prospective cohort of 1006 patients receiving supplementation with 50,000 IU weekly for 128 weeks. Results showed that vitamin D increases were associated with a decrease in disease activity and proteinuria (64). Also, Abou-Raya et al. reported that lower vitamin D levels correlated with disease activity and improvement in inflammatory and hemostatic parameters was observed after 12 months treatment (71). Latest studies have found no association between supplementation and disease activity and no changes in IFN signature in vitamin D deficient SLE patients (72, 73). Other studies have shown effects in cytokine profiles and T cell differentiation (74, 75).

A recent systematic review and meta-analyses of observational and randomized trials (76) found no convincing evidence of a clear role of vitamin D with highly significant results in both randomized and observational studies. The number of randomized, controlled trials with vitamin D is scarce, so more data are needed to reach a conclusion. Also, the effects of multiple compounds when administered simultaneously, and the follow-up time, may be inadequate to allow differences in disease occurrence. More efforts are required in order to clarify the role that vitamin D has in this disease, to regulate the type of supplementation required, and to determine the minimal beneficial levels. Specific attention to maintaining optimal vitamin D levels may be beneficial in the management of SLE.

## Conclusion

Vitamin D exerts important regulatory functions on cells from the innate as well as from the adaptive immune response. Indeed, accumulating evidence has shown that insufficient vitamin D levels may lead to dysregulation of immune responses, and thus contribute to autoimmune diseases. There is no consensus about recommended targeted serum levels and the optimal mode and dose of vitamin D supplementation. It seems that higher doses for supplementation could have better outcomes in disease activity, but still there is great variability between studies and no conclusions can be obtained. More and larger studies are needed to determine how vitamin D supplementation affects the pathophysiology of SLE and how it may contribute to better efficacy of actual therapies.

## Author Contributions

PB, RN, DH, and MI contributed to the design of this work, drafting, and revising it critically. All authors gave their final approval to the manuscript.

## Conflict of Interest Statement

The authors declare that the research was conducted in the absence of any commercial or financial relationships that could be construed as a potential conflict of interest.

## Funding

This work was funded by FONDECYT no. 1141211 (PB and MI) and 1140049 (RN). DH is a CONICYT-PCHA/Doctorado Nacional/2015-21150777 fellow.

## References

[1] Adams JS Hewison M. Update in vitamin D. J Clin Endocrinol Metab (2010) 95(2):471–8. 10.1210/jc.2009-1773 20133466 PMC2840860

[2] Gröber U Spitz J Reichrath J Kisters K Holick MF. Vitamin D: update 2013: from rickets prophylaxis to general preventive healthcare. Dermatoendocrinol (2013) 5(3):331–47. 10.4161/derm.26738 24516687 PMC3908963

[3] Holick MF Vitamin D deficiency. N Engl J Med (2007) 357(3):266–81. 10.1056/NEJMra070553 17634462

[4] Holick MF. Vitamin D: important for prevention of osteoporosis, cardiovascular heart disease, type 1 diabetes, autoimmune diseases, and some cancers. South Med J (2005) 98(10):1024–7. 10.1097/01.SMJ.0000140865.32054.DB 16295817

[5] Holick MF Chen TC Lu Z Sauter E. Vitamin D and skin physiology: a D-lightful story. J Bone Miner Res (2007) 22(Suppl 2):V28–33. 10.1359/jbmr.07s211 18290718

[6] Moan J Porojnicu AC Dahlback A Setlow RB. Addressing the health benefits and risks, involving vitamin D or skin cancer, of increased sun exposure. Proc Natl Acad Sci U S A (2008) 105(2):668–73. 10.1073/pnas.0710615105 18180454 PMC2206594

[7] Holick MF. Vitamin D: a millenium perspective. J Cell Biochem (2003) 88(2):296–307. 10.1002/jcb.10338 12520530

[8] Prietl B Treiber G Pieber TR Amrein K. Vitamin D and immune function. Nutrients (2013) 5(7):2502–21. 10.3390/nu5072502 23857223 PMC3738984

[9] DeLuca HF. Overview of general physiologic features and functions of vitamin D. Am J Clin Nutr (2004) 80(6 Suppl):1689S–96S. 15585789 10.1093/ajcn/80.6.1689S

[10] Carlberg C Campbell MJ. Vitamin D receptor signaling mechanisms: integrated actions of a well-defined transcription factor. Steroids (2013) 78(2):127–36. 10.1016/j.steroids.2012.10.019 23178257 PMC4668715

[11] Yang CY Leung PS Adamopoulos IE Gershwin ME. The implication of vitamin D and autoimmunity: a comprehensive review. Clin Rev Allergy Immunol (2013) 45(2):217–26. 10.1007/s12016-013-8361-3 23359064 PMC6047889

[12] Rosen CJ Gallagher JC. The 2011 IOM report on vitamin D and calcium requirements for North America: clinical implications for providers treating patients with low bone mineral density. J Clin Densitom (2011) 14(2):79–84. 10.1016/j.jocd.2011.03.004 21787514

[13] Ross AC Manson JE Abrams SA Aloia JF Brannon PM Clinton SK The 2011 report on dietary reference intakes for calcium and vitamin D from the Institute of Medicine: what clinicians need to know. J Clin Endocrinol Metab (2011) 96(1):53–8. 10.1210/jc.2010-2704 21118827 PMC3046611

[14] Kongsbak M von Essen MR Levring TB Schjerling P Woetmann A Ødum N Vitamin D-binding protein controls T cell responses to vitamin D. BMC Immunol (2014) 15:35. 10.1186/s12865-014-0035-2 25230725 PMC4177161

[15] Safadi FF Hermey DC Popoff SN Seifert MF. Skeletal resistance to 1,25-dihydroxyvitamin D3 in osteopetrotic rats. Endocrine (1999) 11(3):309–19. 10.1385/ENDO:11:3:309 10786828

[16] Holick MF Siris ES Binkley N Beard MK Khan A Katzer JT Prevalence of vitamin D inadequacy among postmenopausal North American women receiving osteoporosis therapy. J Clin Endocrinol Metab (2005) 90(6):3215–24. 10.1210/jc.2004-2364 15797954

[17] Wortsman J Matsuoka LY Chen TC Lu Z Holick MF. Decreased bioavailability of vitamin D in obesity. Am J Clin Nutr (2000) 72(3):690–3. 10966885 10.1093/ajcn/72.3.690

[18] Dusso AS Brown AJ Slatopolsky E. Vitamin D. Am J Physiol Renal Physiol (2005) 289(1):F8–28. 10.1152/ajprenal.00336.2004 15951480

[19] Grey A Lucas J Horne A Gamble G Davidson JS Reid IR. Vitamin D repletion in patients with primary hyperparathyroidism and coexistent vitamin D insufficiency. J Clin Endocrinol Metab (2005) 90(4):2122–6. 10.1210/jc.2004-1772 15644400

[20] Bischoff-Ferrari HA Shao A Dawson-Hughes B Hathcock J Giovannucci E Willett WC. Benefit-risk assessment of vitamin D supplementation. Osteoporos Int (2010) 21(7):1121–32. 10.1007/s00198-009-1119-3 19957164 PMC3062161

[21] Bolland MJ Bacon CJ Horne AM Mason BH Ames RW Wang TK Vitamin D insufficiency and health outcomes over 5 y in older women. Am J Clin Nutr (2010) 91(1):82–9. 10.3945/ajcn.2009.28424 19906799

[22] Bolland MJ Grey A Avenell A Gamble GD Reid IR. Calcium supplements with or without vitamin D and risk of cardiovascular events: reanalysis of the women’s health initiative limited access dataset and meta-analysis. BMJ (2011) 342:d2040. 10.1136/bmj.d2040 21505219 PMC3079822

[23] Souberbielle JC Body JJ Lappe JM Plebani M Shoenfeld Y Wang TJ Vitamin D and musculoskeletal health, cardiovascular disease, autoimmunity and cancer: recommendations for clinical practice. Autoimmun Rev (2010) 9(11):709–15. 10.1016/j.autrev.2010.06.009 20601202

[24] Lagishetty V Misharin AV Liu NQ Lisse TS Chun RF Ouyang Y Vitamin D deficiency in mice impairs colonic antibacterial activity and predisposes to colitis. Endocrinology (2010) 151(6):2423–32. 10.1210/en.2010-0089 20392825 PMC2875827

[25] Grad R Cod and the consumptive: a brief history of cod-liver oil in the treatment of pulmonary tuberculosis. Pharm Hist (2004) 46(3):106–20. 15712453

[26] Scherberich JE Kellermeyer M Ried C Hartinger A. 1-Alpha-calcidol modulates major human monocyte antigens and toll-like receptors TLR 2 and TLR4 in vitro. Eur J Med Res (2005) 10(4):179–82. 15946915

[27] Steinman RM. Decisions about dendritic cells: past, present, and future. Annu Rev Immunol (2012) 30:1–22. 10.1146/annurev-immunol-100311-102839 22136168

[28] Iruretagoyena MI Wiesendanger M Kalergis AM. The dendritic cell-T cell synapse as a determinant of autoimmune pathogenesis. Curr Pharm Des (2006) 12(2):131–47. 10.2174/138161206775193145 16454731

[29] Gordon JR Ma Y Churchman L Gordon SA Dawicki W. Regulatory dendritic cells for immunotherapy in immunologic diseases. Front Immunol (2014) 5:7. 10.3389/fimmu.2014.00007 24550907 PMC3907717

[30] Adorini L Amuchastegui S Corsiero E Laverny G Le Meur T Penna G. Vitamin D receptor agonists as anti-inflammatory agents. Expert Rev Clin Immunol (2007) 3(4):477–89. 10.1586/1744666X.3.4.477 20477154

[31] Griffin MD Lutz W Phan VA Bachman LA McKean DJ Kumar R. Dendritic cell modulation by 1alpha,25 dihydroxyvitamin D3 and its analogs: a vitamin D receptor-dependent pathway that promotes a persistent state of immaturity in vitro and in vivo. Proc Natl Acad Sci U S A (2001) 98(12):6800–5. 10.1073/pnas.121172198 11371626 PMC34433

[32] Penna G Amuchastegui S Laverny G Adorini L. Vitamin D receptor agonists in the treatment of autoimmune diseases: selective targeting of myeloid but not plasmacytoid dendritic cells. J Bone Miner Res (2007) 22(Suppl 2):V69–73. 10.1359/jbmr.07s217 18290726

[33] Rigby WF Waugh M Graziano RF. Regulation of human monocyte HLA-DR and CD4 antigen expression, and antigen presentation by 1,25-dihydroxyvitamin D3. Blood (1990) 76(1):189–97. 2364169

[34] Rolf L Muris AH Hupperts R Damoiseaux J. Vitamin D effects on B cell function in autoimmunity. Ann N Y Acad Sci (2014) 1317:84–91. 10.1111/nyas.12440 24761763

[35] Chen S Sims GP Chen XX Gu YY Chen S Lipsky PE. Modulatory effects of 1,25-dihydroxyvitamin D3 on human B cell differentiation. J Immunol (2007) 179(3):1634–47. 10.4049/jimmunol.179.3.1634 17641030

[36] Hayes CE Hubler SL Moore JR Barta LE Praska CE Nashold FE. Vitamin D actions on CD4(+) T cells in autoimmune disease. Front Immunol (2015) 6:100. 10.3389/fimmu.2015.00100 25852682 PMC4364365

[37] Croker JA Kimberly RP. SLE: challenges and candidates in human disease. Trends Immunol (2005) 26(11):580–6. 10.1016/j.it.2005.09.001 16168709

[38] Hahn BH Antibodies to DNA. N Engl J Med (1998) 338(19):1359–68. 10.1056/NEJM199805073381906 9571257

[39] Lemire JM Ince A Takashima M. 1,25-Dihydroxyvitamin D3 attenuates the expression of experimental murine lupus of MRL/l mice. Autoimmunity (1992) 12(2):143–8. 10.3109/08916939209150321 1617111

[40] Vaisberg MW Kaneno R Franco MF Mendes NF. Influence of cholecalciferol (vitamin D3) on the course of experimental systemic lupus erythematosus in F1 (NZBxW) mice. J Clin Lab Anal (2000) 14(3):91–6. 10.1002/(SICI)1098-2825(2000)14:3<91::AID-JCLA2>3.0.CO;2-O 10797606 PMC6808021

[41] Linker-Israeli M Elstner E Klinenberg JR Wallace DJ Koeffler HP. Vitamin D(3) and its synthetic analogs inhibit the spontaneous in vitro immunoglobulin production by SLE-derived PBMC. Clin Immunol (2001) 99(1):82–93. 10.1006/clim.2000.4998 11286544

[42] Lerman M Burnham J Behrens E. 1,25 Dihydroxyvitamin D3 limits monocyte maturation in lupus sera. Lupus (2011) 20(7):749–53. 10.1177/0961203310394542 21447602

[43] Ben-Zvi I Aranow C Mackay M Stanevsky A Kamen DL Marinescu LM The impact of vitamin D on dendritic cell function in patients with systemic lupus erythematosus. PLoS One (2010) 5(2):e9193. 10.1371/journal.pone.0009193 20169063 PMC2821911

[44] Wang G Tam LS Kwan BC Li EK Chow KM Luk CC Expression of miR-146a and miR-155 in the urinary sediment of systemic lupus erythematosus. Clin Rheumatol (2012) 31(3):435–40. 10.1007/s10067-011-1857-4 21987229

[45] de Azevêdo Silva J Monteiro Fernandes K Trés Pancotto JA Sotero Fragoso T Donadi EA Crovella S Vitamin D receptor (VDR) gene polymorphisms and susceptibility to systemic lupus erythematosus clinical manifestations. Lupus (2013) 22(11):1110–7. 10.1177/0961203313500549 23945129

[46] Carvalho C Marinho A Leal B Bettencourt A Boleixa D Almeida I Association between vitamin D receptor (VDR) gene polymorphisms and systemic lupus erythematosus in Portuguese patients. Lupus (2015) 24(8):846–53. 10.1177/0961203314566636 25661837

[47] Mao S Huang S. Association between vitamin D receptor gene BsmI, FokI, ApaI and TaqI polymorphisms and the risk of systemic lupus erythematosus: a meta-analysis. Rheumatol Int (2014) 34(3):381–8. 10.1007/s00296-013-2898-6 24212677

[48] Xiong J He Z Zeng X Zhang Y Hu Z. Association of vitamin D receptor gene polymorphisms with systemic lupus erythematosus: a meta-analysis. Clin Exp Rheumatol (2014) 32(2):174–81. 24321519

[49] Ruiz-Irastorza G Egurbide MV Olivares N Martinez-Berriotxoa A Aguirre C. Vitamin D deficiency in systemic lupus erythematosus: prevalence, predictors and clinical consequences. Rheumatology (Oxford) (2008) 47(6):920–3. 10.1093/rheumatology/ken121 18411213

[50] Yap KS Morand EF. Vitamin D and systemic lupus erythematosus: continued evolution. Int J Rheum Dis (2015) 18(2):242–9. 10.1111/1756-185X.12489 25522756

[51] Ritterhouse LL Crowe SR Niewold TB Kamen DL Macwana SR Roberts VC Vitamin D deficiency is associated with an increased autoimmune response in healthy individuals and in patients with systemic lupus erythematosus. Ann Rheum Dis (2011) 70(9):1569–74. 10.1136/ard.2010.148494 21586442 PMC3149865

[52] Wright TB Shults J Leonard MB Zemel BS Burnham JM. Hypovitaminosis D is associated with greater body mass index and disease activity in pediatric systemic lupus erythematosus. J Pediatr (2009) 155(2):260–5. 10.1016/j.jpeds.2009.02.033 19446841

[53] Kamen DL Cooper GS Bouali H Shaftman SR Hollis BW Gilkeson GS. Vitamin D deficiency in systemic lupus erythematosus. Autoimmun Rev (2006) 5(2):114–7. 10.1016/j.autrev.2005.05.009 16431339

[54] Huisman AM White KP Algra A Harth M Vieth R Jacobs JW Vitamin D levels in women with systemic lupus erythematosus and fibromyalgia. J Rheumatol (2001) 28(11):2535–9. 11708429

[55] Mok CC. Vitamin D and systemic lupus erythematosus: an update. Expert Rev Clin Immunol (2013) 9(5):453–63. 10.1586/eci.13.19 23634739

[56] Costenbader KH Feskanich D Holmes M Karlson EW Benito-Garcia E. Vitamin D intake and risks of systemic lupus erythematosus and rheumatoid arthritis in women. Ann Rheum Dis (2008) 67(4):530–5. 10.1136/ard.2007.072736 17666449 PMC2717608

[57] Hiraki LT Munger KL Costenbader KH Karlson EW. Dietary intake of vitamin D during adolescence and risk of adult-onset systemic lupus erythematosus and rheumatoid arthritis. Arthritis Care Res (Hoboken) (2012) 64(12):1829–36. 10.1002/acr.21776 22744978 PMC3488139

[58] Mok CC Birmingham DJ Ho LY Hebert LA Song H Rovin BH. Vitamin D deficiency as marker for disease activity and damage in systemic lupus erythematosus: a comparison with anti-dsDNA and anti-C1q. Lupus (2012) 21(1):36–42. 10.1177/0961203311422094 21993384

[59] Mok CC Birmingham DJ Leung HW Hebert LA Song H Rovin BH. Vitamin D levels in Chinese patients with systemic lupus erythematosus: relationship with disease activity, vascular risk factors and atherosclerosis. Rheumatology (Oxford) (2012) 51(4):644–52. 10.1093/rheumatology/ker212 21719424

[60] Lertratanakul A Wu P Dyer A Urowitz M Gladman D Fortin P 25-Hydroxyvitamin D and cardiovascular disease in patients with systemic lupus erythematosus: data from a large international inception cohort. Arthritis Care Res (Hoboken) (2014) 66(8):1167–76. 10.1002/acr.22291 24470118 PMC4844829

[61] Mandal M Tripathy R Panda AK Pattanaik SS Dakua S Pradhan AK Vitamin D levels in Indian systemic lupus erythematosus patients: association with disease activity index and interferon alpha. Arthritis Res Ther (2014) 16(1):R49. 10.1186/ar4479 24507879 PMC3979045

[62] Schoindre Y Jallouli M Tanguy ML Ghillani P Galicier L Aumaître O Lower vitamin D levels are associated with higher systemic lupus erythematosus activity, but not predictive of disease flare-up. Lupus Sci Med (2014) 1(1):e000027. 10.1136/lupus-2014-000027 25379192 PMC4213833

[63] Bogaczewicz J Sysa-Jedrzejowska A Arkuszewska C Zabek J Kontny E McCauliffe D Vitamin D status in systemic lupus erythematosus patients and its association with selected clinical and laboratory parameters. Lupus (2012) 21(5):477–84. 10.1177/0961203311427549 22065093

[64] Petri M Bello KJ Fang H Magder LS. Vitamin D in systemic lupus erythematosus: modest association with disease activity and urine protein/creatinine ratio. Arthritis Rheum (2013) 65(7):1865–71. 10.1002/art.37953 23553077 PMC3701725

[65] Reynolds JA Haque S Berry JL Pemberton P Teh LS Ho P 25-Hydroxyvitamin D deficiency is associated with increased aortic stiffness in patients with systemic lupus erythematosus. Rheumatology (Oxford) (2012) 51(3):544–51. 10.1093/rheumatology/ker352 22120462 PMC3281497

[66] Toloza SM Cole DE Gladman DD Ibañez D Urowitz MB. Vitamin D insufficiency in a large female SLE cohort. Lupus (2010) 19(1):13–9. 10.1177/0961203309345775 19897520

[67] Souto M Coelho A Guo C Mendonça L Argolo S Papi J Vitamin D insufficiency in Brazilian patients with SLE: prevalence, associated factors, and relationship with activity. Lupus (2011) 20(10):1019–26. 10.1177/0961203311401457 21646315

[68] Stockton KA Kandiah DA Paratz JD Bennell KL. Fatigue, muscle strength and vitamin D status in women with systemic lupus erythematosus compared with healthy controls. Lupus (2012) 21(3):271–8. 10.1177/0961203311425530 22004972

[69] Schneider L Colar da Silva AC Werres Junior LC Alegretti AP Dos Santos AS Santos M Vitamin D levels and cytokine profiles in patients with systemic lupus erythematosus. Lupus (2015) 24(11):1191–7. 10.1177/0961203315584811 25926056

[70] Ruiz-Irastorza G Gordo S Olivares N Egurbide MV Aguirre C. Changes in vitamin D levels in patients with systemic lupus erythematosus: effects on fatigue, disease activity, and damage. Arthritis Care Res (Hoboken) (2010) 62(8):1160–5. 10.1002/acr.20186 20235208

[71] Abou-Raya A Abou-Raya S Helmii M. The effect of vitamin D supplementation on inflammatory and hemostatic markers and disease activity in patients with systemic lupus erythematosus: a randomized placebo-controlled trial. J Rheumatol (2013) 40(3):265–72. 10.3899/jrheum.111594 23204220

[72] Andreoli L Dall’Ara F Piantoni S Zanola A Piva N Cutolo M A 24-month prospective study on the efficacy and safety of two different monthly regimens of vitamin D supplementation in pre-menopausal women with systemic lupus erythematosus. Lupus (2015) 24(4–5):499–506. 10.1177/0961203314559089 25801893

[73] Aranow C Kamen DL Dall’Era M Massarotti EM Mackay MC Koumpouras F Randomized, double-blind, placebo-controlled trial of the effect of vitamin D3 on the interferon signature in patients with systemic lupus erythematosus. Arthritis Rheumatol (2015) 67(7):1848–57. 10.1002/art.39108 25777546 PMC4732716

[74] Piantoni S Andreoli L Scarsi M Zanola A Dall’Ara F Pizzorni C Phenotype modifications of T-cells and their shift toward a Th2 response in patients with systemic lupus erythematosus supplemented with different monthly regimens of vitamin D. Lupus (2015) 24(4–5):490–8. 10.1177/0961203314559090 25801892

[75] Terrier B Derian N Schoindre Y Chaara W Geri G Zahr N Restoration of regulatory and effector T cell balance and B cell homeostasis in systemic lupus erythematosus patients through vitamin D supplementation. Arthritis Res Ther (2012) 14(5):R221. 10.1186/ar4060 23075451 PMC3580532

[76] Theodoratou E Tzoulaki I Zgaga L Ioannidis JP. Vitamin D and multiple health outcomes: umbrella review of systematic reviews and meta-analyses of observational studies and randomised trials. BMJ (2014) 348:g2035. 10.1136/bmj.g2035 24690624 PMC3972415

